# Supplementary material for: Dynamic birefringence and chirality of magnetically controllable assemblies of anisotropic plasmonic nanoparticles in dispersion
Source: Nat Commun. 2025 Aug 1;16:7076. doi: 10.1038/s41467-025-62508-0 (PMC12316907; doi:10.1038/s41467-025-62508-0)
Supplement: Supplementary file 1 — Supplementary Information [file 41467_2025_62508_MOESM1_ESM.pdf]

Supplementary Information

Dynamic birefringence and chirality of magnetically controllable assemblies of anisotropic plasmonic nanoparticles in dispersion

Hyojung Kang[1], Yoojung Jeon[1], Kyungnae Baek[1], SeonJu Park[1], Jayoon Lee[1],
Tae Soup Shim[2,3], Jerome K. Hyun[1] & So-Jung Park*[1,4]*

1 Department of Chemistry and Nanoscience, Ewha Womans University, 52 Ewhayeodae-gil, Seodaemun-gu, Seoul 03760, Korea

2 Department of Energy Systems Research, Ajou University, 206 World cup-ro, Yeongtong-gu, Suwon 16499, Korea

3 Department of Chemical Engineering, Ajou University, 206 World cup-ro, Yeongtong-gu, Suwon 16499, Korea

4 Graduate Program in Innovative Biomaterials Convergence, Ewha Womans University, Seoul 03760, Korea

* E-mail: kadam.hyun@ewha.ac.kr; sojungpark@ewha.ac.kr

Supplementary Note 1. Redox reactions involved in FeOOH formation.

The formation of FeOOH on the AuNR surface occurs mainly through the following redox reactions.

$$2Fe^{2+} + PtCl_4^{2-} + 4H_2O \rightarrow 2FeOOH(s) + Pt(s) + 4Cl^- + 4H^+ \quad (1)$$

$$4Fe^{2+} + O_2 + 6H_2O \rightarrow 4FeOOH(s) + 8H^+ \quad (2)$$

Supplementary Table 1. Finite-difference Time-domain (FDTD) simulation parameters. Geometrical parameters of AuNS/Fe_xO_y/SiO_2 (1) and AuNR/Fe_xO_y/SiO_2 (2-7) structures used in FDTD simulations.

Sample No.	Width of AuNR (nm)	Length of AuNR (nm)	Aspect ratio	Thickness of Fe_xO_y (nm)	Thickness of SiO_2 (nm)
1	40	40	1.0	3.0	20
2	40	60	1.5	3.0	15
3	18	32	1.8	3.0	20
4	35	77	2.2	3.0	25
5	30	69	2.3	3.0	22
6	30	81	2.7	2.8	25
7	18	61	3.4	3.0	25

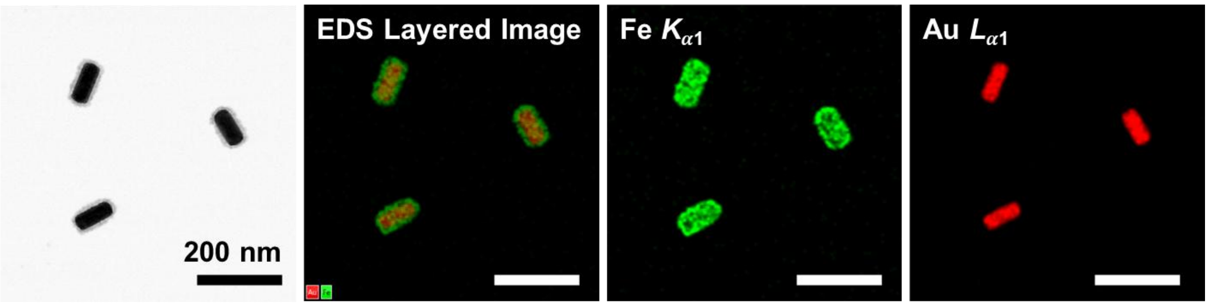

Supplementary Fig. 1. Elemental analysis of FeOOH-coated AuNRs. Transmission electron microscopy (TEM) and Energy-dispersive X-ray spectroscopy (EDS) mapping images of FeOOH-coated AuNRs

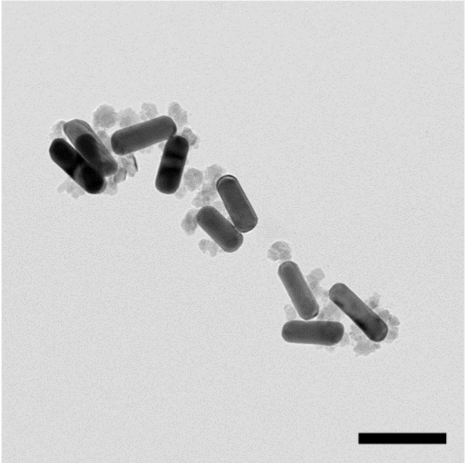

Supplementary Fig. 2. Effect of K_2PtCl_4. TEM image of FeOOH-coated AuNRs synthesized without K_2PtCl_4. The scale bar indicates 100 nm.

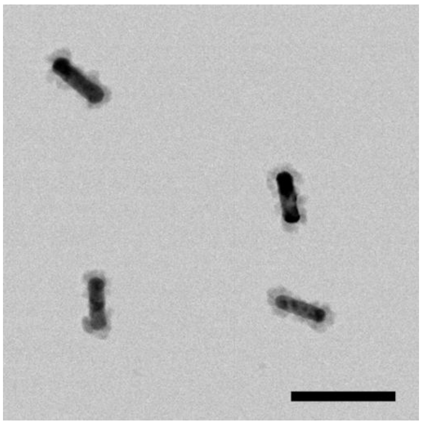

Supplementary Fig. 3. Effect of AuNR concentration. TEM image of FeOOH-coated AuNRs synthesized at a higher concentration of AuNRs (6.6 nM). The $FeCl_2 \cdot 4H_2O$ concentration was 10 mM. The scale bar indicates 100 nm.

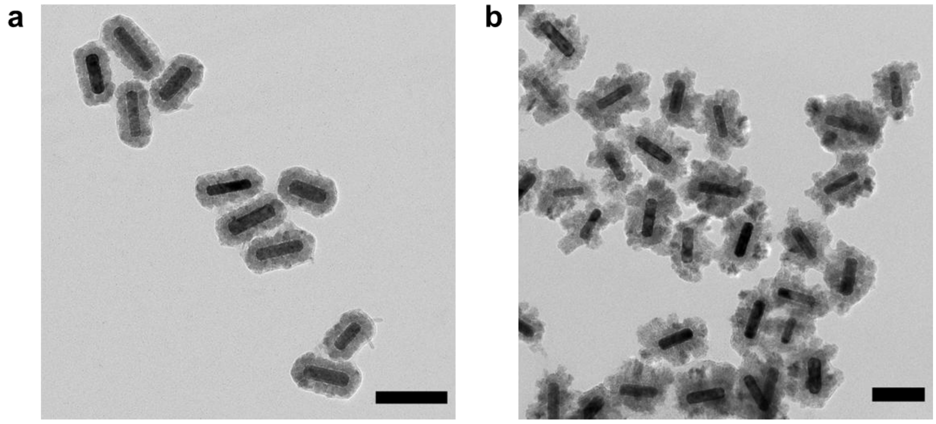

Supplementary Fig. 4. Effect of $FeCl_2 \cdot 4H_2O$ concentration. TEM images of FeOOH-coated AuNRs synthesized with varying amounts of $FeCl_2 \cdot 4H_2O$ (20 mM (a) and 200 mM (b)). The K_2PtCl_4 and AuNR concentrations were 0.4 mM and 4.4 nM, respectively. The scale bars indicate 100 nm for all images.

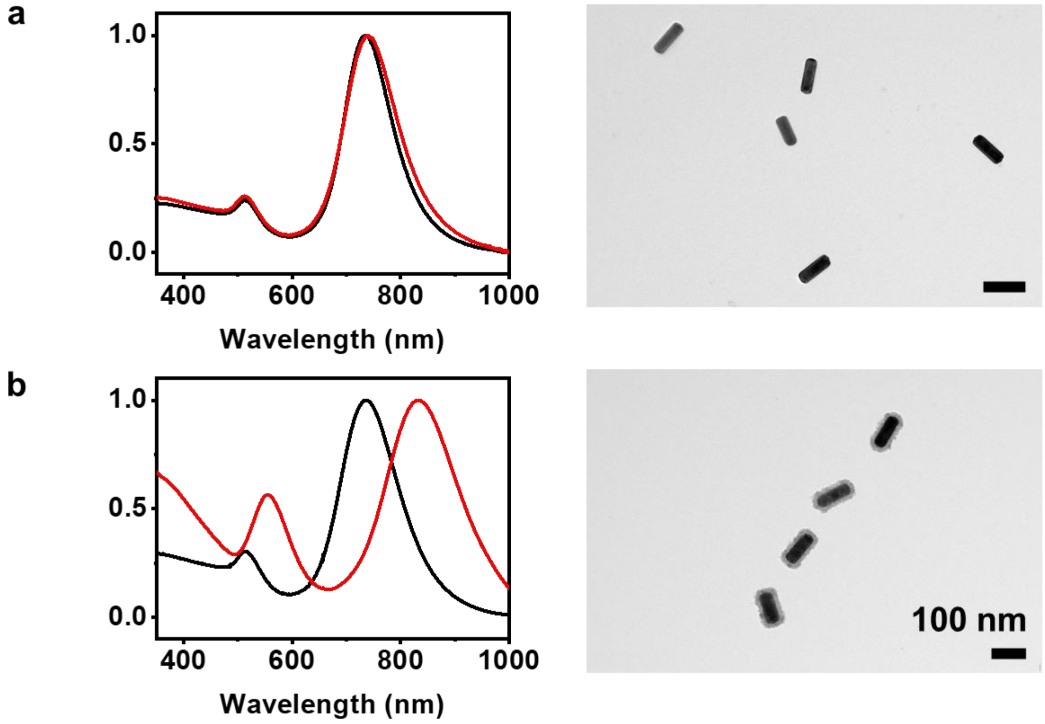

Supplementary Fig. 5. Effect of surfactants. Extinction spectra (black: as-synthesized AuNR, red: AuNR subjected to FeOOH coating procedure) and TEM images of AuNRs subjected to FeOOH coating procedure under (a) hexadecyltrimethylammonium bromide (CTAB, i.e., without surfactant change) and (b) hexadecyltrimethylammonium chloride (CTAC) conditions.

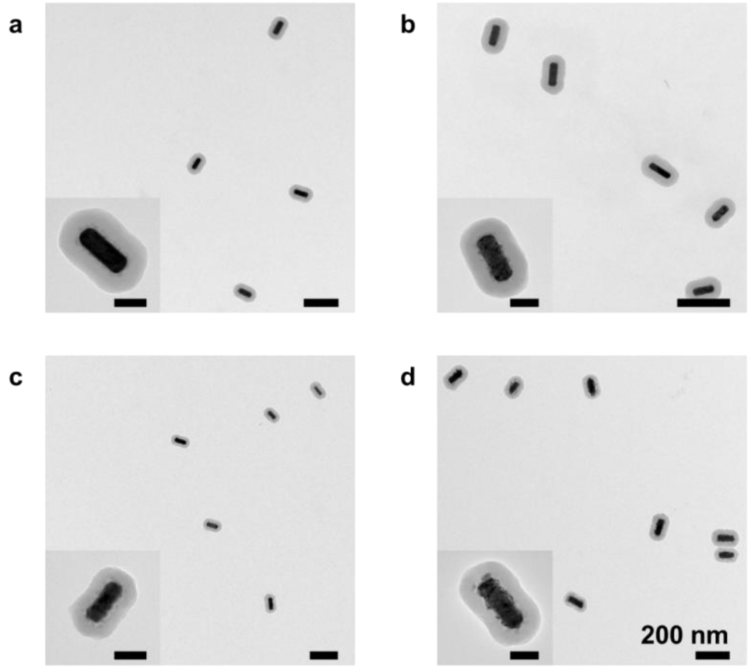

Supplementary Fig. 6. Effect of reduction conditions. TEM images of magnetically controllable plasmonic nanoparticles (MPs) reduced at (a) 260 °C for 30 min, (b) 310 °C for 30 min, (c) 360 °C for 30 min, and (d) 360 °C for 2 h. The inset scale bars indicate 50 nm for all images.

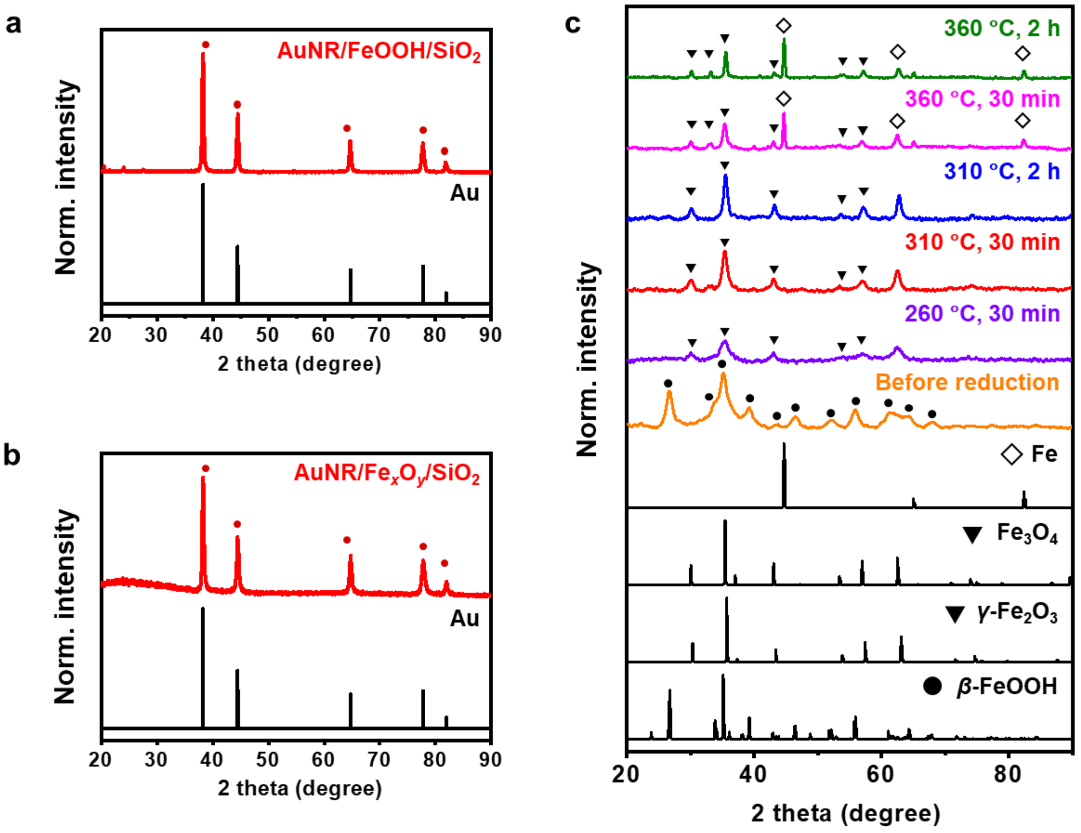

Supplementary Fig. 7. X-ray diffraction (XRD) analysis. XRD profiles of (a) AuNR/FeOOH/SiO_2, (b) AuNR/Fe_xO_y/SiO_2 (reduction condition of 310 °C and 2 h). The XRD patterns of AuNR/FeOOH/SiO_2 and AuNR/Fe_xO_y/SiO_2 were dominated by the strong gold signals, making it difficult to analyze the porous iron-containing layer. (c) XRD profiles of FeOOH (before reduction) and its reduction products formed at different conditions (temperature and duration). Black lines are literature data from the Crystallography Open Database (COD).

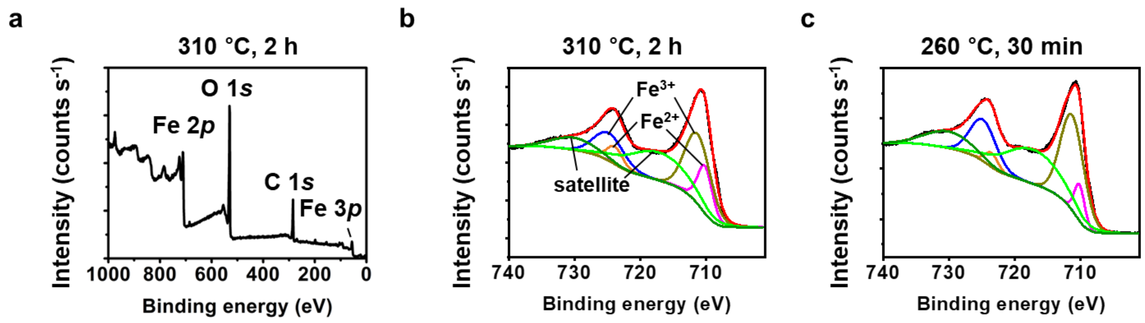

Supplementary Fig. 8. X-ray photoelectron spectroscopy (XPS) analysis. (a) XPS spectra of Fe_xO_y prepared by reducing FeOOH at 310 °C for 2 h, showing the presence of Fe 2*p*, O 1*s*, C 1*s*, and Fe 3*p* peaks with binding energies at 711, 530, 284, and 56 eV, respectively. (b-c) The deconvoluted Fe 2*p* spectra of Fe_xO_y prepared by reducing FeOOH at (b) 310 °C for 2 h or (c) 260 °C for 30 min displays peaks at 711 eV (Fe $2p_{3/2}$) and 724 eV (Fe $2p_{1/2}$). Fe 2*p* spectra were deconvoluted into characteristic peaks, with fitted positions at 711.3 eV (Fe^{3+} $2p_{3/2}$), 710.2 eV (Fe^{2+} $2p_{3/2}$), 724.8 eV (Fe^{3+} $2p_{1/2}$), and 723.5 eV (Fe^{2+} $2p_{1/2}$).

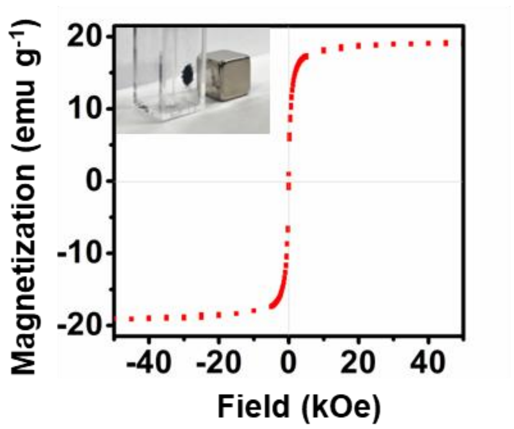

Supplementary Fig. 9. Magnetization behavior. Magnetization curve of MPs measured at 300 K. The inset image shows the magnetic response of MPs in the powder form.

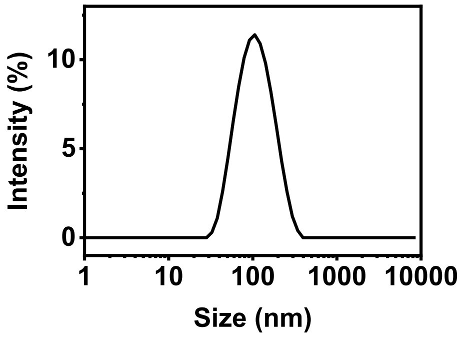

Supplementary Fig. 10. Hydrodynamic size distribution. Dynamic light scattering (DLS) data of MPs after dispersion in water by brief sonication. The measured diameter was 95.7 nm with dispersity in size (Đ) of 0.188.

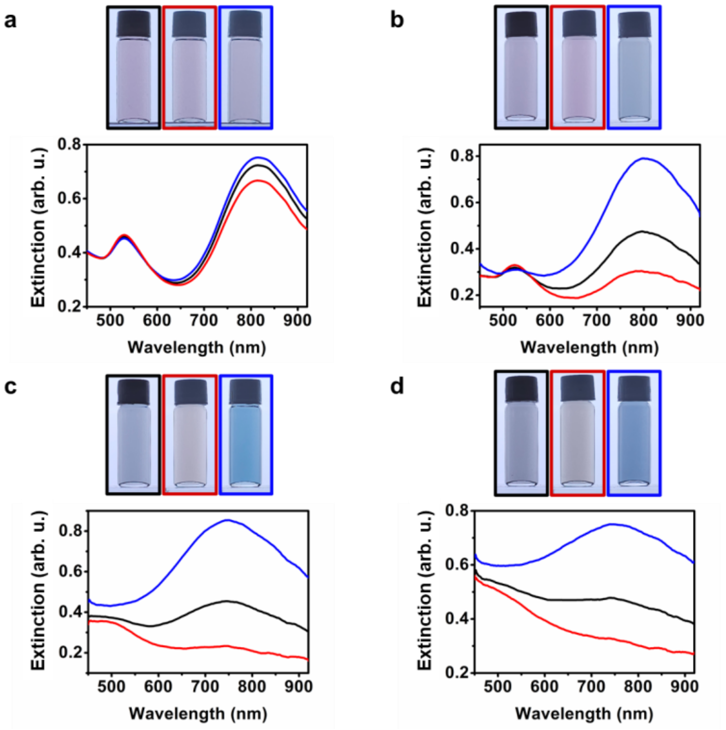

Supplementary Fig. 11. Magneto-optical response of MPs formed under varying reduction conditions. Photographs and extinction spectra collected under B_x (18 mT) for MPs prepared at reduction conditions of (a) 260 °C for 30 min, (b) 310 °C for 30 min, (c) 360 °C for 30 min, and (d) 360 °C for 2 h. The spectra and photographs were acquired under unpolarized light (black), polarized light parallel to the magnetic field direction ($P_{\parallel}$, blue), and polarized light perpendicular to the magnetic field direction ($P_{\perp}$, red).

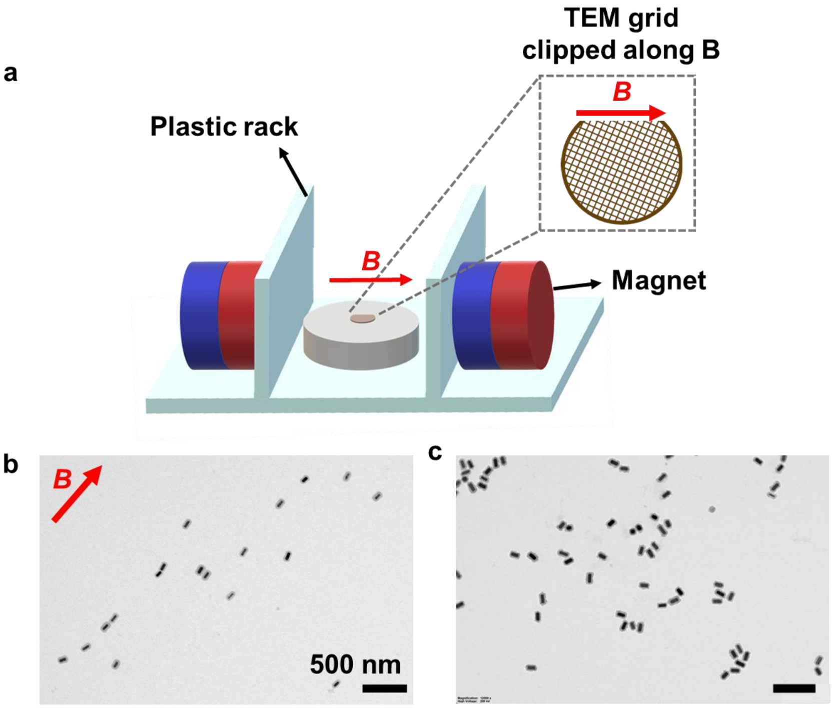

Supplementary Fig. 12. Alignment of MPs under a magnetic field. (a) Schematic illustration of the experimental setup for linear magnetic field generation for TEM sampling. TEM images of MPs (b) with and (c) without an external magnetic field (18 mT). A droplet of MP solution was placed on a TEM grid between a pair of cylindrical NdFeB magnets (3.8 cm diameter × 1.9 cm thick, grade N52, K & J Magnetics, Inc.) separated by a plastic rack (end-to-end distance of 18 cm), which was then left overnight for drying. The upper edge of the TEM grid was cut along the direction of the applied magnetic field to mark the field direction.

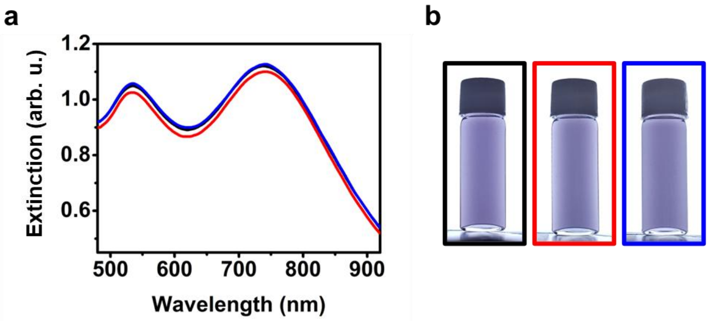

Supplementary Fig. 13. Optical property of MPs in the absence of an external magnetic field. Extinction spectra (a) and photographs (b) of an MP solution under unpolarized light (black), $P_{\parallel}$ (blue), and $P_{\perp}$ (red) without an external magnetic field.

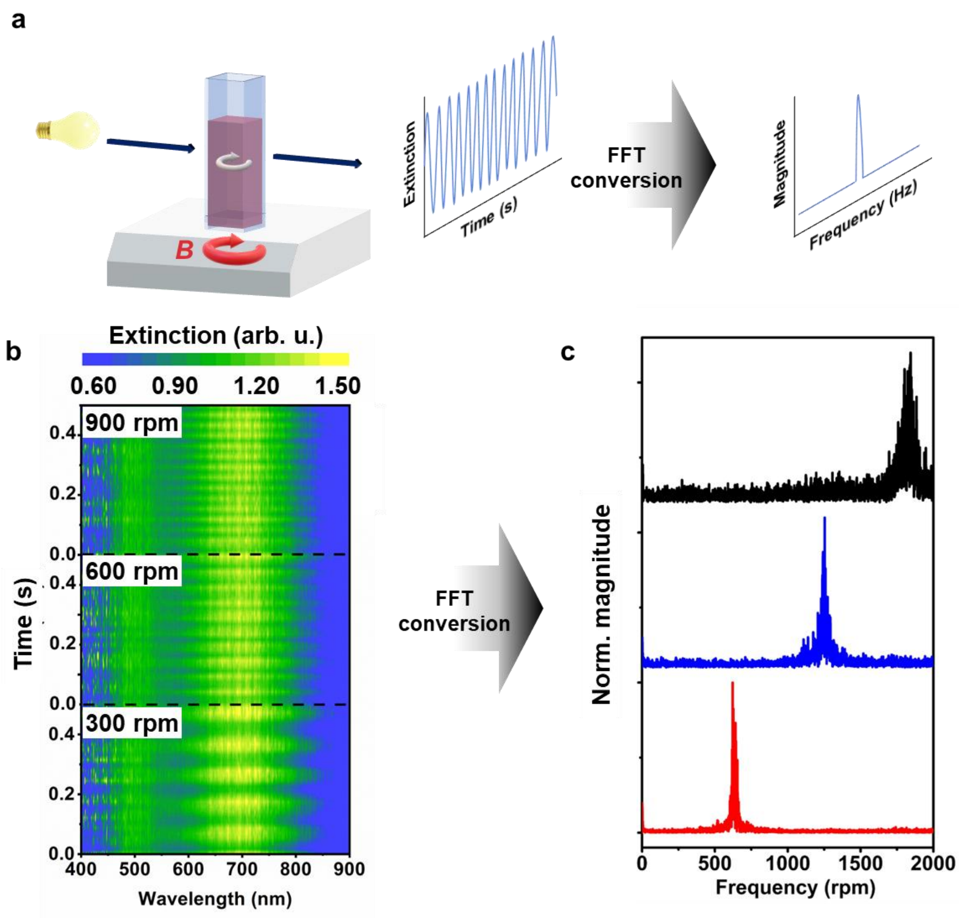

Supplementary Fig. 14. Optical modulation under a rotating magnetic field. (a) The measurement set-up of the optical response under a rotating magnetic field. (b) Time-resolved extinction map of MPs under different rotating speeds of the magnetic field. The color bar on top represents the extinction intensity. (c) Frequency peak after fast Fourier transform (FFT) conversion under a rotating magnetic field at 300 rpm (red), 600 rpm (blue), and 900 rpm (black). The signal intensity of the MPs was measured at the longitudinal localized surface plasmon resonance (LSPR) peak position at the interval of 15 ms. The FFT generated frequency peaks at 600, 1200, and 1800 rpm respectively, which correspond to the doubling of the rotating speed.

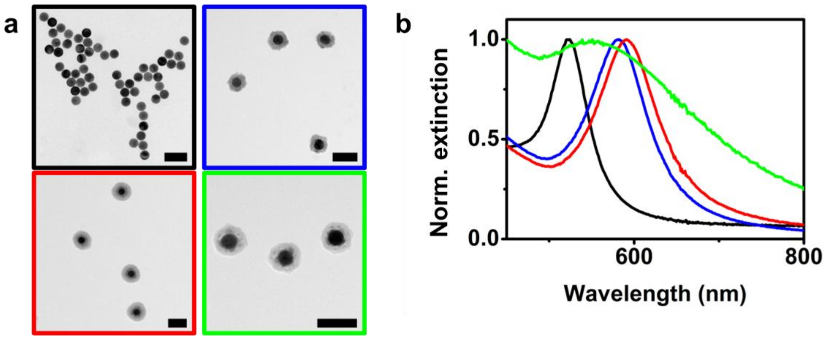

Supplementary Fig. 15. Characterization of AuNS/Fe$_x$O$_y$/SiO$_2$. (a) TEM images and (b) extinction spectra of AuNSs (black), AuNS/FeOOH (blue), AuNS/FeOOH/SiO$_2$ (red), and AuNS/Fe$_x$O$_y$/SiO$_2$ (green). The scale bars indicate 100 nm for all images.

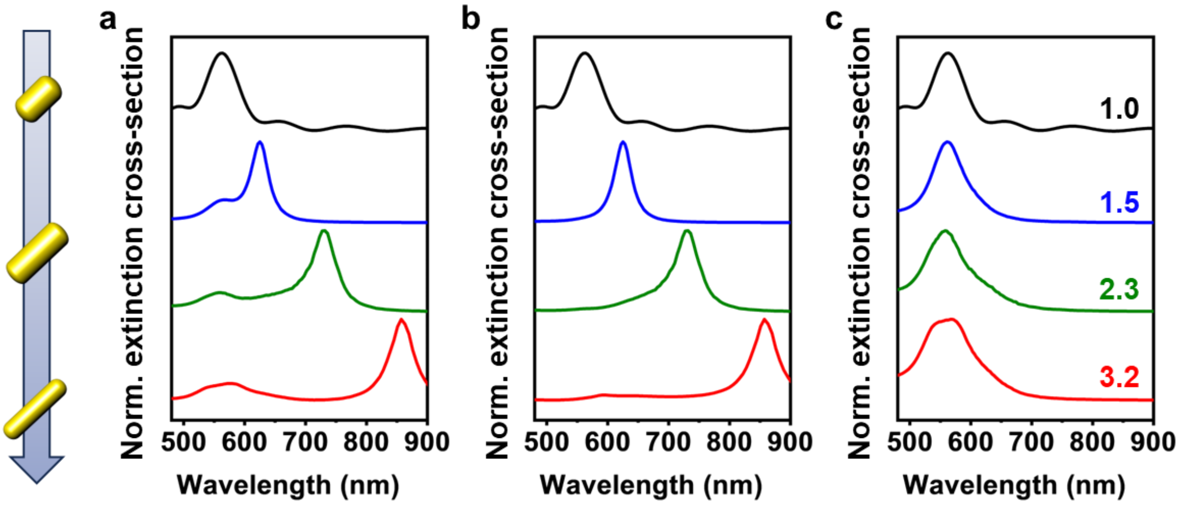

Supplementary Fig. 16. Simulated extinction spectra. Simulated extinction cross-section of AuNS/Fe$_x$O$_y$/SiO$_2$ (black) and AuNR/Fe$_x$O$_y$/SiO$_2$ with AuNR aspect ratios of 1.5 (blue), 2.3 (green), and 3.2 (red) under an external magnetic field with (a) unpolarized light, (b) $P_{\parallel}$, and (c) $P_{\perp}$.

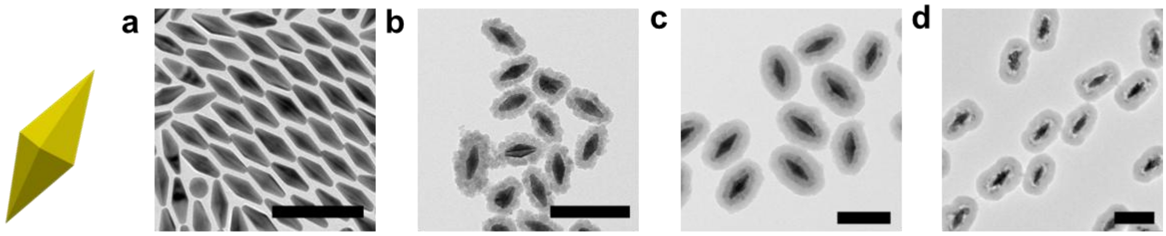

Supplementary Fig. 17. Synthesis of AuNBP/Fe$_x$O$_y$/SiO$_2$. TEM images of (a) AuNBPs, (b) AuNBP/FeOOH, (c) AuNBP/FeOOH /SiO$_2$, and (d) AuNBP/Fe$_x$O$_y$/SiO$_2$. The scale bars indicate 100 nm for all images.

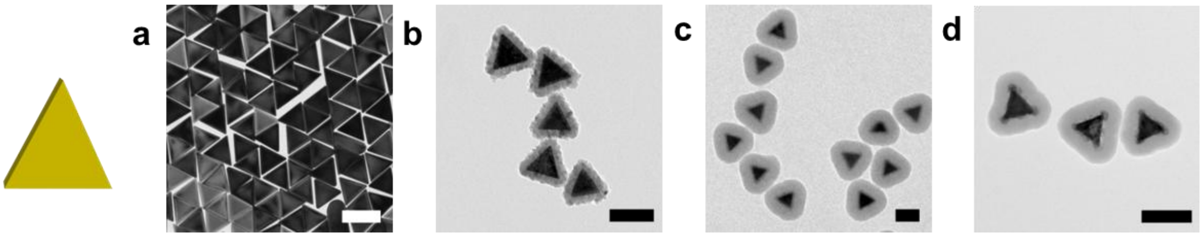

Supplementary Fig. 18. Synthesis of AuNT/Fe$_x$O$_y$/SiO$_2$. TEM images of (a) AuNTs, (b) AuNT/FeOOH, (c) AuNT/FeOOH/SiO$_2$, and (d) AuNT/Fe$_x$O$_y$/SiO$_2$. The scale bars indicate 100 nm for all images.

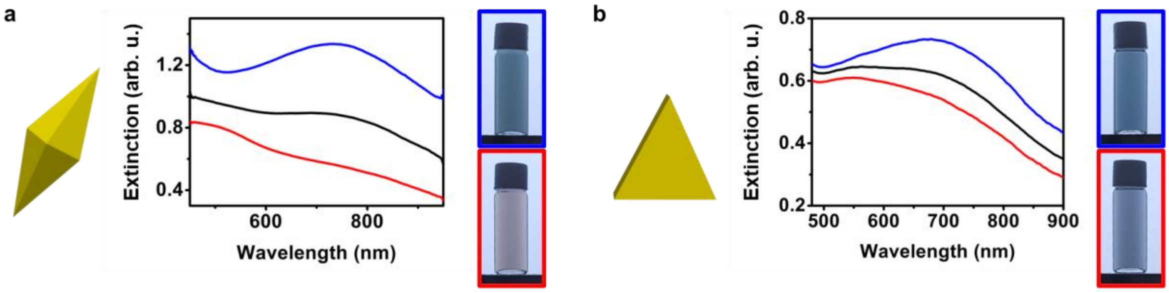

Supplementary Fig. 19. Optical properties of AuNBP/Fe$_x$O$_y$/SiO$_2$ and AuNT/Fe$_x$O$_y$/SiO$_2$. Extinction spectra and photographs of (a) AuNBP/Fe$_x$O$_y$/SiO$_2$ and (b) AuNT/Fe$_x$O$_y$/SiO$_2$ under an external magnetic field (18 mT) with unpolarized light (black), $P_{\parallel}$ (blue), and $P_{\perp}$ (red).

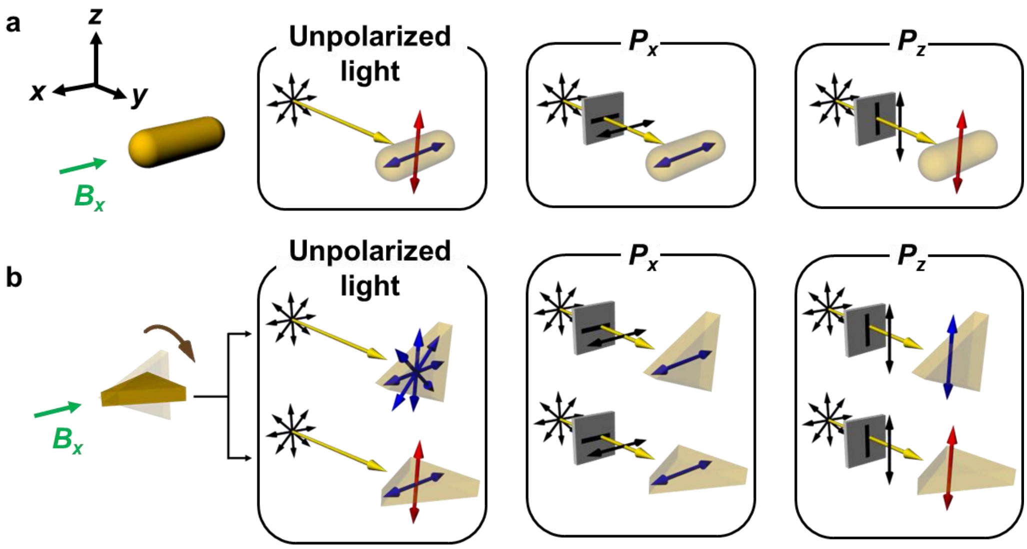

Supplementary Fig. 20. Illustration of NP orientation and LSPR modes under B_x. Schematic illustration of NP orientation in (a) 1D and (b) 2D structures under B_x. Green arrows indicate the magnetic field direction. Black arrows indicate the polarization direction of incident light. Blue and red arrows represent the longitudinal LSPR (L-LSPR) and transverse LSPR (T-LSPR) modes in (a), and the in-plane and out-of-plane LSPR modes in (b), respectively.

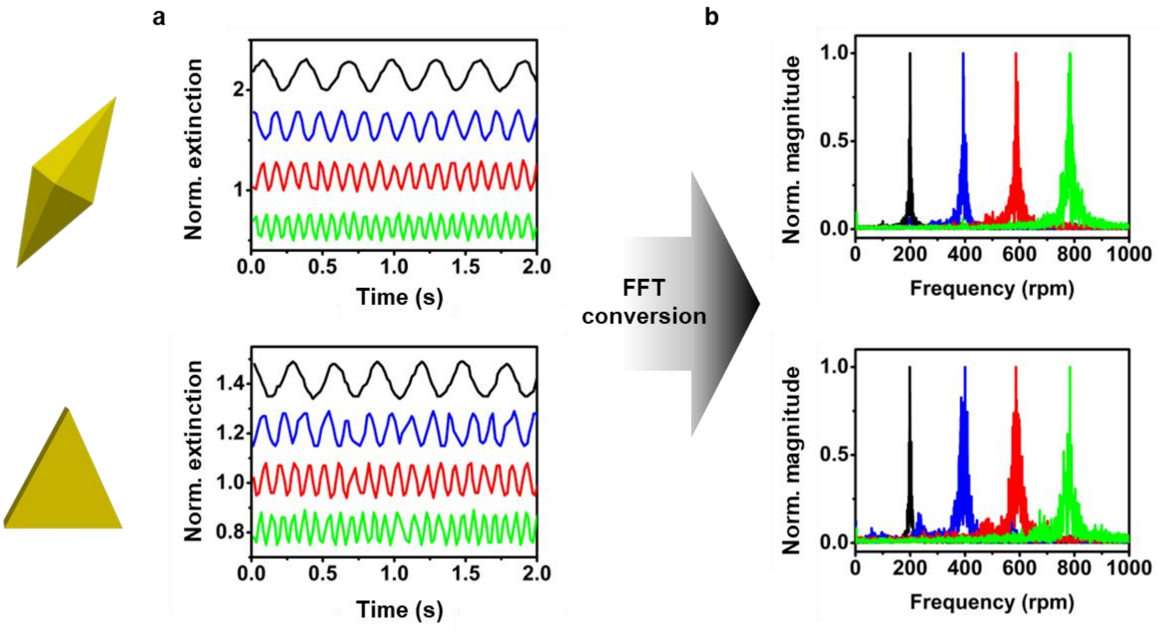

Supplementary Fig. 21. Optical response under rotating magnetic fields. (a) Optical response as a function of time and (b) FFT frequency shift after FFT conversion of AuNBP/Fe_xO_y/SiO_2 (top) and AuNT/Fe_xO_y/SiO_2 (bottom) under a rotating magnetic field at 100 rpm (black), 200 rpm (blue), 300 rpm (red), and 400 rpm (green). The signal intensity was measured at the L-LSPR peak for AuNBP/Fe_xO_y/SiO_2 and the in-plane mode for AuNT/Fe_xO_y/SiO_2 at the interval of 15 ms.

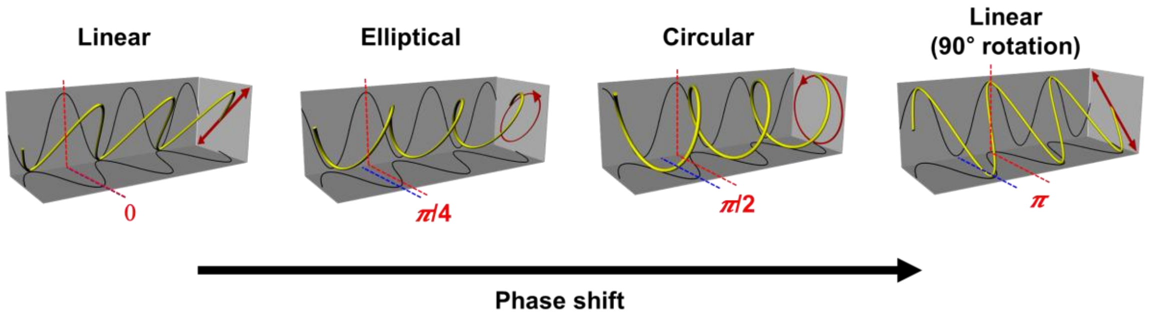

Supplementary Fig. 22. Illustration of polarization rotation induced by a phase shift. The incident electric field (yellow curve) is composed of two orthogonal components (black curves). Their superposition gives rise to elliptical, circular, or linear polarization states, depending on the relative phase shift—$\pi/4$, $\pi/2$, and π, respectively. At a phase shift of π, the resulting polarization returns to a linear state, rotated by 90° with respect to the original polarization direction.

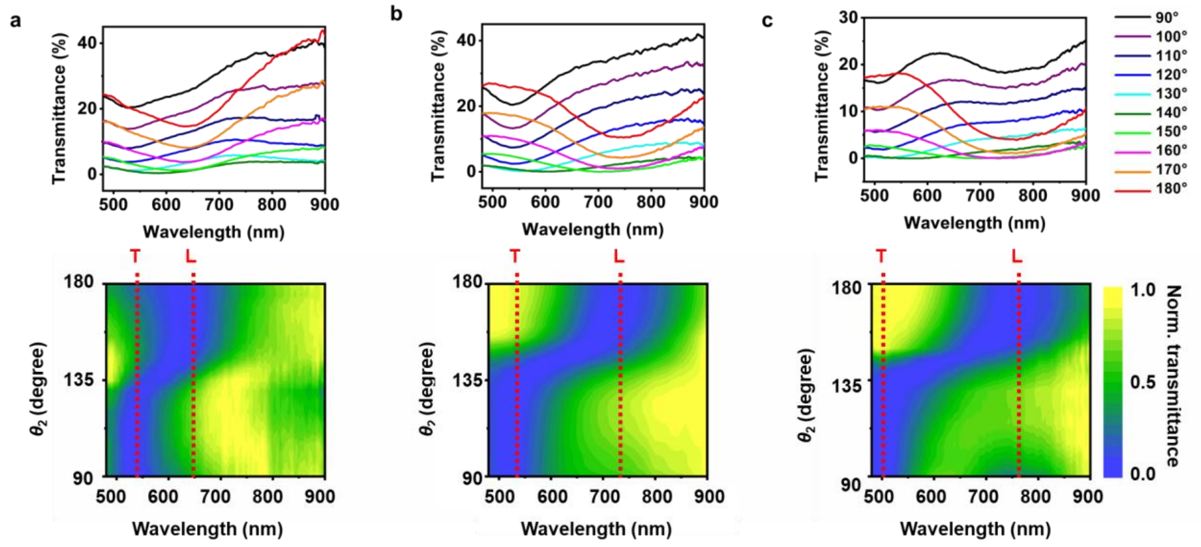

Supplementary Fig. 23. Transmittance spectra for MPs with varying AuNR aspect ratios. Transmission spectra of MPs containing AuNRs with aspect ratios of (a) 1.9, (b) 2.3, and (c) 3.4 under B_x (18 mT), $\theta_1 = 45°$, and $\theta_2 = 90°–180°$.

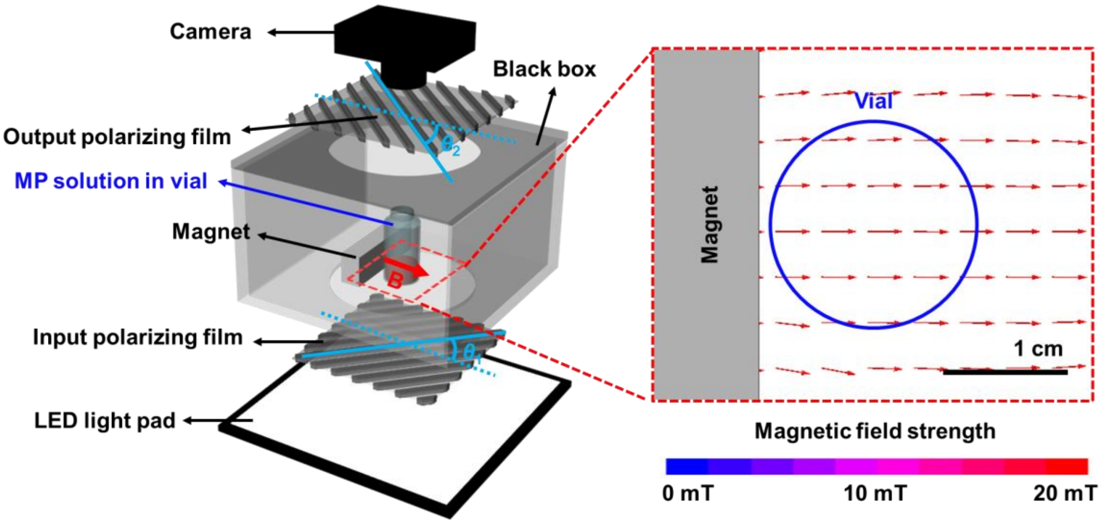

Supplementary Fig. 24. Optical set-up for imaging birefringent colors under a magnetic field. Schematic illustration of the setup for optical imaging of MP solutions under a magnetic field and input/output polarizers. A bar-shaped NdFeB magnet (gray block) is positioned adjacent to a vial containing the MP solution to apply the linear magnetic field (18 mT). The red arrow indicates the magnetic field direction. The inset (red box) presents the magnetic field distribution calculated by Ansys Maxwell Electromechanical Device Analysis Software, confirming the linear magnetic field at the sample position (inside the blue circle). The magnetic field intensity is represented by the color scale bar. The input (θ_1) and output (θ_2) polarizer angles are defined relative to the applied magnetic field direction (blue dotted line).

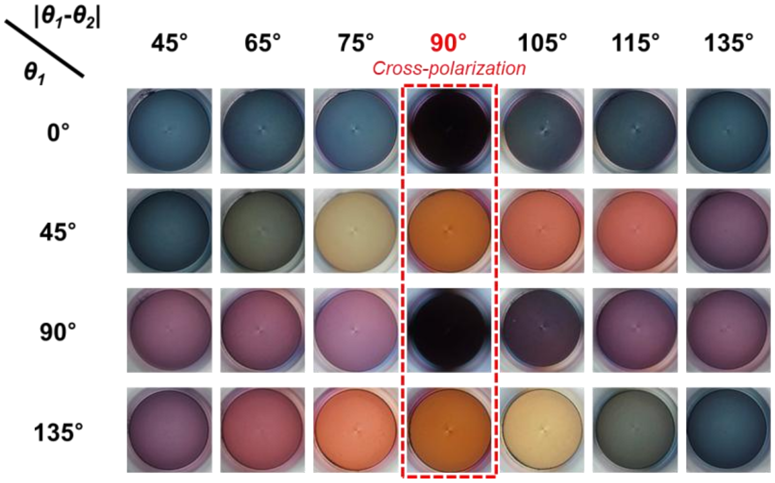

Supplementary Fig. 25. Solution colors under varying polarizer angles. Photographs of MP (AuNR aspect ratio: 2.3) solutions under the linear B_x (18 mT) with varying θ_1 and θ_2.

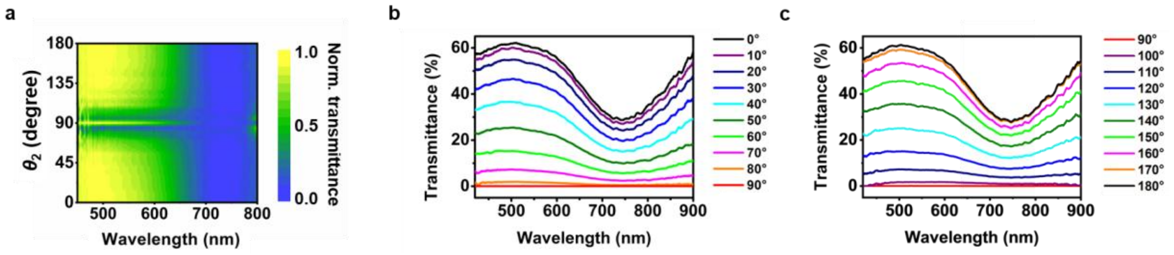

Supplementary Fig. 26. Transmittance spectra at θ_1 = 0°. (a) Transmission plot (0° < θ_2 < 180°, 5° steps) and (b, c) transmission spectra of MPs (AuNR aspect ratio: 2.3) under B_x (18 mT) at θ_1 = 0° and varying θ_2 (b: θ_2 = 0°–90°, c: θ_2 = 90°–180°).

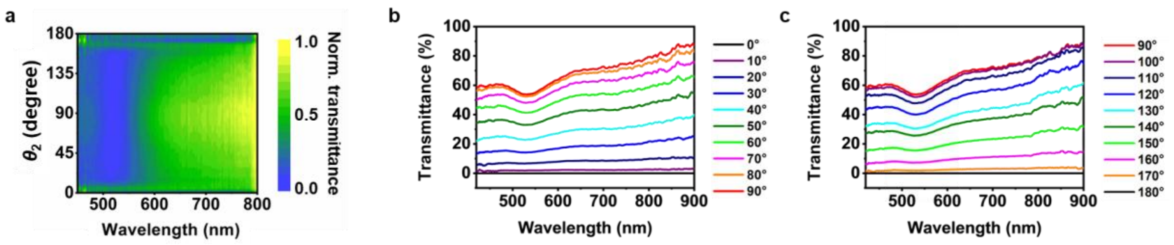

Supplementary Fig. 27. Transmittance spectra at θ_1 = 90° (a) Transmission plot (0° < θ_2 < 180°, 5° steps) and (b, c) transmission spectra of MPs (AuNR aspect ratio: 2.3) under B_x (18 mT) at θ_1 = 90° and varying θ_2.

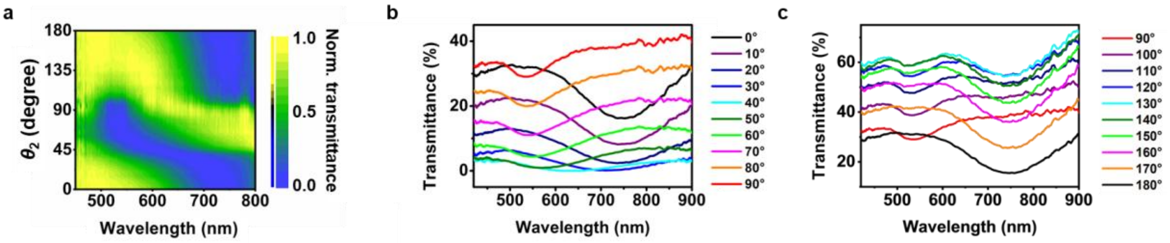

Supplementary Fig. 28. Transmittance spectra at $\theta_1 = 135°$ (a) Transmission plot ($0° < \theta_2 < 180°$, 5° steps) and (b, c) transmission spectra of MPs (AuNR aspect ratio: 2.3) under B_x (18 mT) at θ_1 = 135° and varying θ_2.

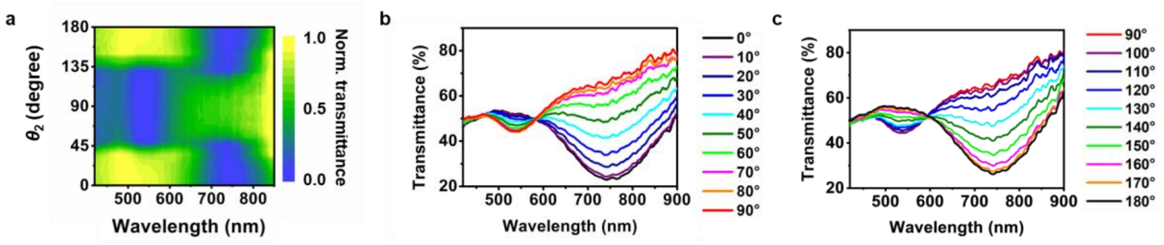

Supplementary Fig. 29. Transmittance spectra without an input polarizer. (a) Transmission plot ($0° < \theta_2 < 180°$, 5° steps) and (b, c) transmission spectra of MPs (AuNR aspect ratio: 2.3) under B_x (18 mT) at varying θ_2 without an input polarizer.

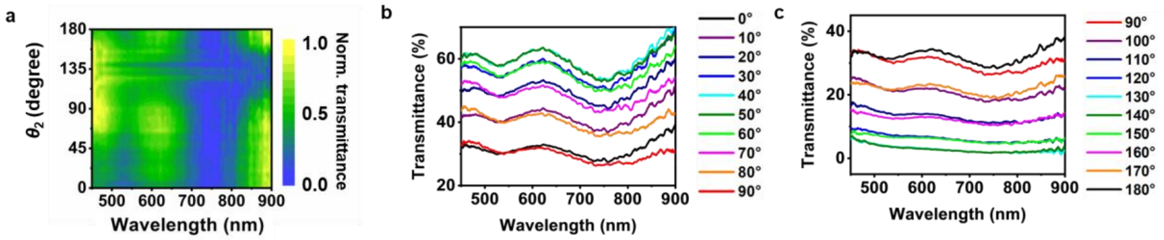

Supplementary Fig. 30. Transmittance spectra in the absence of a magnetic field. (a) Transmission plot ($0° < \theta_2 < 180°$, 5° steps) and (b, c) transmission spectra of MPs (AuNR aspect ratio: 2.3) at θ_1 = 45° and varying θ_2 without a magnetic field.

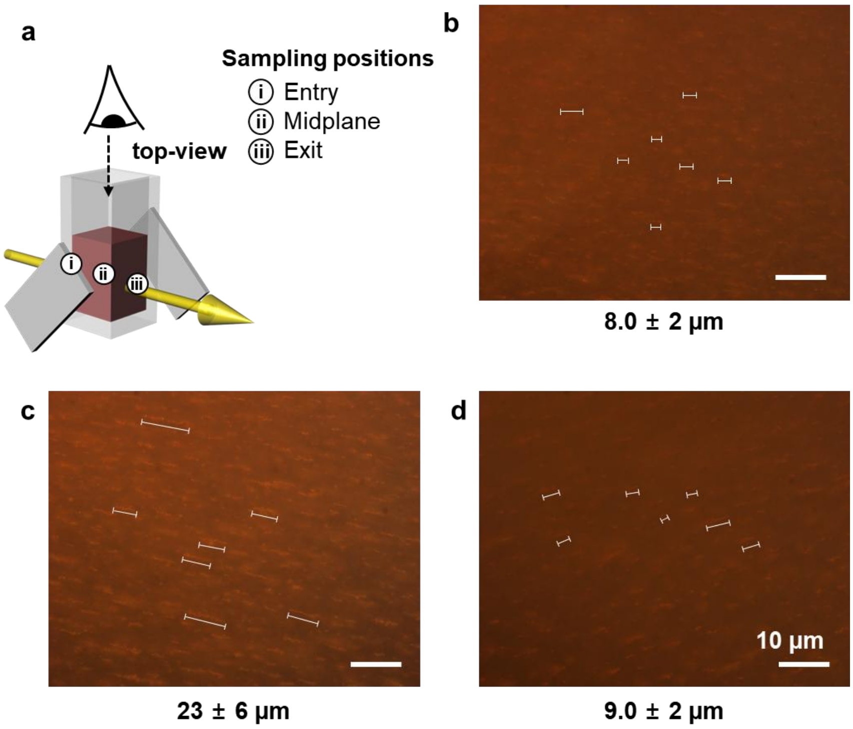

Supplementary Fig. 31. Helical field-induced alignment of MPs. Reflection-mode optical microscopy analysis of MP alignment under a helical magnetic field. (a) Schematic of the optical path and three sampling positions across the cuvette (① Entry, ② Midplane, ③ Exit). (b–d) Reflection-mode dark-field optical images of MP assemblies fixed in a hydrogel at each position. The projected lengths of the assemblies are shortest at the entry (b) and exit (d), and longest at the midplane (c), indicating a progressive axial tilting of the particle chains along the light propagation direction.

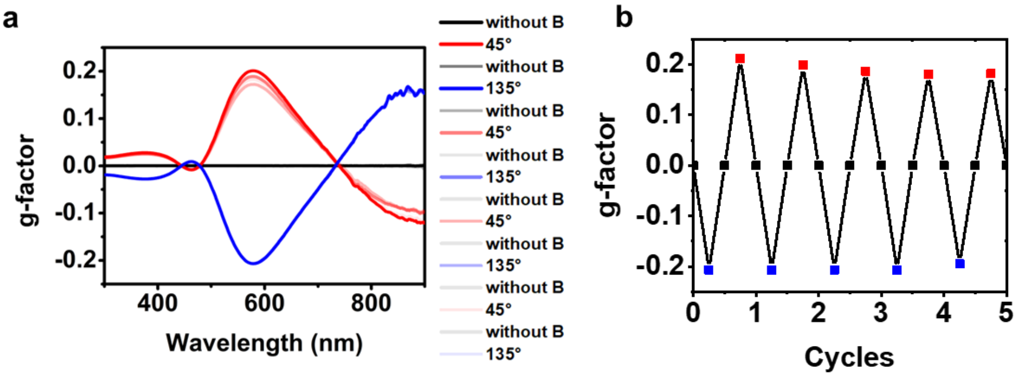

Supplementary Fig. 32. Reversibility of chiroptical responses of MPs. (a) *g*-factor spectra and (b) *g*-factor values under repeated alternating helical magnetic fields (20 mT) generated using the cross-bar magnet set-up described in Figure 5a (left-handed helical field: red, right-handed helical field: blue, no magnetic field: black).

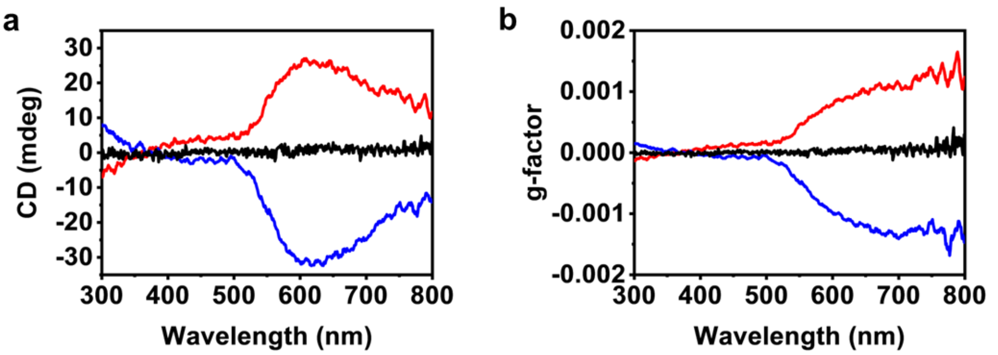

Supplementary Fig. 33. Chiroptical responses of AuNS-based MPs. (a) Circular dichroism (CD) spectra and (b) *g*-factor spectra of AuNS/Fe_xO_y/SiO_2 under helical magnetic fields (20 mT) with 45° (red) or 135° (blue) magnet settings (Figure 5a,b). The data collected without a magnetic field are presented in black for comparison.

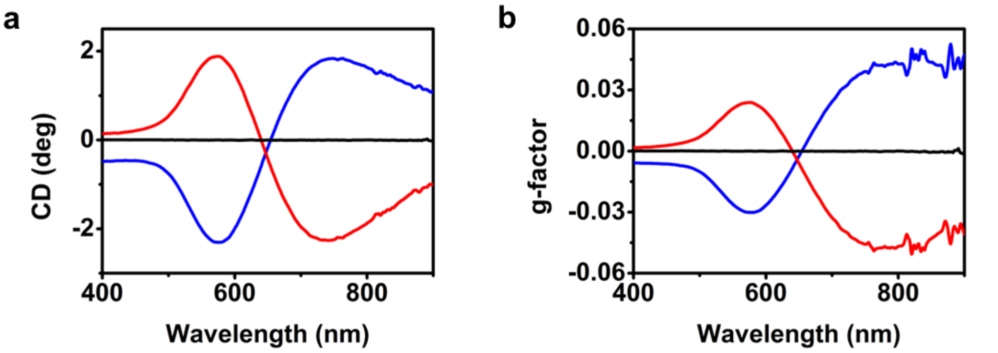

Supplementary Fig. 34. Chiroptical responses of MPs with an AuNR aspect ratio of 1.9. (a) CD spectra and (b) *g*-factor spectra of MPs containing AuNRs with an aspect ratio of 1.9 under helical magnetic fields (20 mT) with 45° (red) or 135° (blue) magnet settings (Figure 5a,b). The data collected without a magnetic field are presented in black for comparison.
