# Supplementary material for: Dynamic birefringence and chirality of magnetically controllable assemblies of anisotropic plasmonic nanoparticles in dispersion
Source: Nat Commun. 2025 Aug 1;16:7076. doi: 10.1038/s41467-025-62508-0 (PMC12316907; doi:10.1038/s41467-025-62508-0)
Supplement: Supplementary file 2 — Description of Additional Supplementary Files [file 41467_2025_62508_MOESM2_ESM.pdf]

Description of Additional Supplementary Files

File Name: Supplementary Movie 1

Description: Dispersion of MPs in water by brief sonication.

File Name: Supplementary Movie 2

Description: Simulated magnetic field distribution under a helical magnetic field ($\theta = 45°$).

File Name: Supplementary Movie 3

Description: Simulated magnetic field distribution under a helical magnetic field ($\theta = 135°$).
